# True polyandry and pseudopolyandry: why does a monandrous fly remate?

**DOI:** 10.1186/1471-2148-13-157

**Published:** 2013-07-25

**Authors:** David N Fisher, Rowan J Doff, Tom A R Price

**Affiliations:** 1 Centre for Ecology and Conservation, University of Exeter, Penryn Campus, Treliever Road, Penryn TR10 9EZ, UK; 2 Institute of Integrative Biology, University of Liverpool, Biosciences Building, Crown Street, Liverpool L69 7ZB, UK

**Keywords:** Copulation duration, Infertility, Monandry, Plasticity, Polyandry, Sperm competition, Social environment

## Abstract

**Background:**

The rate of female remating can have important impacts on a species, from affecting conflict and cooperation within families, to population viability and gene flow. However, determining the level of polyandry in a species can be difficult, with information on the mating system of many species being based on a single experiment, or completely absent. Here we investigate the mating system of the fruit fly *Drosophila subobscura*. Reports from England, Spain and Canada suggest *D. subobscura* is entirely monandrous, with no females remating. However, work in Greece suggests that 23% of females remate. We examine the willingness of female *D. subobscura* to remate in the laboratory in a range of conditions, using flies from both Greece and England. We make a distinction between pseudopolyandry, where a female remates after an ineffective first mating that is incapable of fertilising her eggs, and true polyandry, where a female remates even though she has received suitable sperm from a previous mating.

**Results:**

We find a low rate of true polyandry by females (4%), with no difference between populations. The rate of true polyandry is affected by temperature, but not starvation. Pseudopolyandry is three times as common as true polyandry, and most females showing pseudopolyandry mated at their first opportunity after their first failed mating. However, despite the lack of differences in polyandry between the populations, we do find differences in the way males respond to exposure to other males prior to mating. In line with previous work, English flies responded to one or more rivals by increasing their copulation duration, a response previously thought to be driven by sperm competition. Greek males only show increased copulation duration when exposed to four or more rival males. This suggests that the response to rivals in *D. subobscura* is not related to sperm competition, because sperm competition is rare, and there is no correlation of response to rivals and mating system across the populations.

**Conclusions:**

These results illustrate the difficulties in determining the mating system of a species, even one that is well known and an excellent laboratory species, with results being highly dependent on the conditions used to assay the behaviour, and the population used.

## Background

Female remating rate is extremely variable, ranging from females mating with a single male in their lifetime, to extreme polyandry where a female may mate with hundreds of males [1,2]. These differences in mating system can have profound effects on a species, impacting on effective population size and population viability [3], the rate of gene flow [4], and the spread of sexually transmitted [5,6] and genetic diseases [7]. Female remating rate also impacts on relatedness within families and hence the level of conflict, cooperation, and sociality within family groups [8,9]. The mating system has a particularly strong impact on the degree of pre and post-copulatory choice by females [10], competition between males [11], male–female conflict [12], and subsequent adaptations to these pressures [13,14].

However, despite the importance of polyandry and the decades of research into its causes and consequences, there is still considerable confusion over polyandry in the literature. The use of the word “polyandry” varies between papers, and difficulties in determining the level of polyandry in a species further confuse the issue. Some authors have used the term polyandry for instances where a female has remated after receiving sperm incapable of fertilising her e.g. [2,15-17] and thus where sperm competition cannot be taking place. However, we argue that almost all the key ecological and evolutionary impacts of polyandry (see previous paragraph) depend on the possibility of a female’s offspring being sired by multiple fathers [18]. In the many cases where offspring cannot be fathered by a male (due to the copulation being a short pseudocopulation without sperm transfer, male sterility, or genetic incompatibility preventing fertilisation) the consequences of the mating are greatly reduced. Only the spread of sexually transmitted diseases, the risk of mechanical damage to male or female, and prevention of infanticide by males will be affected equally by fertile and infertile copulations. In species where males provide resources to females during or after mating, such as nuptial gifts or parental care, females may gain from infertile matings with additional males. However, high costs to males are expected to drive choosiness and high fertility in males, potentially reducing the likelihood of infertile matings in such species. Despite these potential costs and benefits of infertile matings, we argue that the presence of sperm competition is the factor that makes the most important difference between polyandry and monandry (generally defined as “females mating with a single male”), and that it would be useful to clarify the terminology around polyandry to reflect this.

We suggest the convention that “true polyandry” refers to females mating with more than one male after which sperm competition occurs. In contrast, “pseudopolyandry” refers to females mating with more than one male where sperm competition does not occur. This convention would leave “polyandry” as a general term covering all situations where a female mates with multiple males. Throughout this paper, this is the convention we will use. Obviously in many cases it will be difficult to distinguish true polyandry from pseudopolyandry. Nevertheless, we think it is important for biologists to have clearly defined concepts to use if their data allow it.

Even when using the broadest sense of monandry and polyandry, determining the mating system of a species is often difficult. Typically the mating system of a species is determined using a relatively small number of observations, either in the field or laboratory, or with paternity analysis using genetic techniques [19,20], often from a single location and time point [21]. However, the number of times a female mates may vary between populations [22], environmental conditions [23], and both the physical condition [24,25] and genotype [26-28] of the female. As a very simple example, if a particular population has an unusually short female lifespan, it may be impossible for a normally polyandrous species to mate more than once, forcing it to become monandrous under those circumstances. As a result, many species referred to as “polyandrous” or “monandrous” may show considerable variation, and in many cases the assigned mating system may simply be wrong.

Here we investigate the mating system of a well-studied species, the fruit fly *D. subobscura*, a model species for research into evolution [29], genetics [30], ecology [31] and climate change [32]. However, despite decades of research there is still considerable confusion about the mating system in this species. It has generally been thought to be completely monandrous, with evidence coming from studies conducted on populations from England [33], Spain [34] and Canada [35]. However, a study on a population from Greece detected insemination by at least two males in about 20% of wild-caught and mass laboratory reared females, with the authors estimating that all wild females may mate multiple times [36]. Further evidence suggesting this species is polyandrous comes from a study on male investment in mating after exposure to a potential rival male. Lizé and colleagues [35] found that male *D. subobscura* that were reared with one or more males had longer copulation durations than males reared in isolation. This increase in copulation duration when males are exposed to potential rivals is seen in many *Drosophila* species [35,37] and has been interpreted as a response to the increased risk of sperm competition. Work on *D. melanogaster*[37] and *D. pseudoobscura*[38] has shown that this increase in copulation effort increases offspring production and success in sperm competition. In *D. pseudoobscura*, males exposed to rivals increase the number of fertilising sperm transferred to the female [38]. In *D. melanogaster* males exposed to rivals transfer more sperm to females, but only if their hearing and taste senses function normally [39]. In D*. melanogaster* the transcription and transfer of seminal fluid proteins also increases after exposure to rivals [40], although there is some evidence that actual production of seminal proteins may drop [41]. If the response of *D. subobscura* males to rivals is a response to sperm competition, it suggests that polyandry must be present in many populations.

We tested for remating in *D. subobscura* in two populations, one from the UK (thought to be monandrous) and one from Greece (thought to be polyandrous), under a range of laboratory conditions. As *D. subobscura* males often feed females prior to mating [42,43], we included low nutrition conditions. We also manipulated male exposure to potential rival males, and observed their subsequent mating behaviour to test whether there is variation in the response to rivals in this species, and whether this correlates with polyandry as predicted by theory [37]. In line with our definitions above, we classified females as showing true polyandry when they remated after a fertile mating (one that produced offspring). If a female remated after producing no offspring from her first mating, we classified this as pseudopolyandry. See methods for further details.

## Results

See Table 1 for the percentages of females showing true polyandry, pseudopolyandry, and overall remating rate in each of the different conditions.

**Table 1:** Female remating behaviour of the two populations in different conditions

**Condition**	**Population**	**N**	**True polyandry**	**Pseudopolyandry**	**All rematings**
Standard	UK	124	4.0% (5)	9.7% (12)	15.3% (19)
	Greek	118	3.4% (4)	9.3% (11)	13.6% (16
♂ from other population	UK	77	5.2% (4)	10.4% (8)	19.5% (15)
	Greek	102	4.9% (5)	8.8% (9)	14.7% (17)
♂& ♀ stored at 18°C, mating took place at 18°C	UK	43	0.0% (0)	11.6% (5)	11.6% (5)
	Greek	34	5.9% (2)	8.8% (3)	20.6% (7)
♂& ♀ stored at 25°C, mating took place at 25°C	UK	36	13.9% (5)	8.3% (3)	30.6% (11)
	Greek	30	6.7% (2)	16.7% (5)	30.0% (9)
♂& ♀ stored at18°C, mating took place at 21°C	UK	21	0.0% (0)	19.0% (4)	19.0% (4)
	Greek	16	0.0% (0)	6.3% (1)	6.3% (1)
♂& ♀ stored at 25°C, mating took place at 21°C	UK	24	4.2% (1)	8.3% 2)	12.5% (3)
	Greek	18	0.0% (0)	5.6% (1)	11.1% (2)
♂ stored at 25°C, ♀ stored at 18°C, mating took place at 21°C	UK	22	0.0% (0)	4.5% (1)	13.6% (3)
	Greek	24	12.5% (3)	16.7% (4)	29.2% (7)
♂ stored at 18°C, ♀ stored at 25°C, mating took place at 21°C	UK	18	0.0% (0)	5.6% (1)	5.6% (1)
	Greek	20	5.0% (1)	0.0% (0)	5.0% (1)
♂ starved	UK	56	3.6% (2)	10.7% (6)	17.9% (10)
	Greek	45	2.2% (1)	15.6% (7)	20.0% (9)
♀ starved	UK	17	0.0% (0)	11.8% 2)	11.8% (2)
	Greek	-	-	-	-
♂ &♀ starved	UK	31	6.5% (2)	12.9% (4)	19.4% (6)
	Greek	-	-	-	-
All	All	893	4.1% (37)	10.3% (92)	14.4% (152)

The percentage of females showing true polyandry (remating after a fertile first mating), pseudopolyandry (remating after an infertile first mating) and all rematings, for flies from two populations under a variety of experimental conditions. Numbers in brackets are the actual number of females that remated. Standard conditions were females mated with a male from the same population, reared and mated at 21°C, with ample food. Note that the number of all rematings can be greater than pseudo- and true polyandry combined, as females whose mating status could not be determined were still observed to remate. The data for conditions involving starved Greek females is not shown as few mated initially (N = 8 for starved female, fed males, and N = 9 for starved females, starved males), preventing analysis of remating behaviour.

### Population differences in mating rate

The population of the female did not affect the rate of true polyandry (GLM, χ^2^_1, 372_ =0.004, p = 0.948), and neither did the population of the male in the first mating (GLM, χ^2^_1, 372_ = 0.034, p = 0.855) nor the interaction between male and female populations (GLM, χ^2^_1, 372_ = 0.464, p = 0.496). The same was true when examining pseudopolyandry, (GLM, population of the female: χ^2^_1, 374_ = 0.020, p = 0.887; male: χ^2^_1, 373_ = 0.023, p = 0.879; interaction: χ^2^_1, 372_ = 0.002, p = 0.966). No factors significantly predicted the number of days until remating, either for true polyandry (p > 0.648 in all cases) or pseudopolyandry (p > 0.278 in all cases).

### Impact of temperature on remating

The rate of true polyandry was affected by a significant interaction between the temperature the female was kept at and the population of the cross (GLM, χ^2^_1, 394_ = 4.246, p = 0.039). The rate of true polyandry in UK flies showed a linear increase with increasing temperature, whereas for the Greek flies there was a non-significant U-shaped trend, with the lowest levels of polyandry at 21°C. UK flies showed significant differences in rate of true polyandry at different female storage temperatures (0%, 4.4% and 8.3% at 18°C, 21°C and 25°C respectively; Pearson’s Chi-squared test, χ^2^_2_ = 6.247, p = 0.044) driven by a difference between the 18 and 25°C treatments (Pearson’s Chi-squared test with Yates’ continuity correction, 18–21 p = 0.171, 18–25 p = 0.034, 21–25 p = 0.437). This difference in true polyandry was not detected in the Greek flies (7.5%, 3.8% and 5.7% at 18°C, 21°C and 25°C respectively; Pearson’s Chi-squared test, χ^2^_2_ = 1.064, p = 0.588). There was a trend for the rate of true polyandry to increase at higher mating temperatures, but this was marginally non-significant (GLM, χ^2^_1, 394_ = 3.429, p = 0.064). The temperature the male was kept at did not significantly predict rate of true polyandry (GLM, χ^2^_1, 394_ = 0.400, p = 0.527). All other factors were non-significant (p >0.121 in all cases).

The rate of pseudopolyandry was affected by an interaction between the temperature the male was kept at and the population of the cross, although this was very marginally non-significant (GLM, χ^2^_1, 479_ = 3.816, p = 0.051). Greek females tended to be more likely to be pseudopolyandrous if the male was stored at a higher temperature (5.7%, 9.3% and 13.9% at 18°C, 21°C and 25°C respectively), while UK females showed the reverse trend of being less pseudopolyandrous if the male was kept at a higher temperature (12.2%, 9.7% and 7.3% at 18°C, 21°C and 25°C respectively). If the interaction is considered non-significant, then none of the other factors affected the rate of pseudopolyandry (GLM, female storage temperature: χ^2^_1, 478_ = 0.778, p = 0.378; male storage temperature: χ^2^_1, 481_ = 0.137, p = 0.712; temperature the mating occurred at: χ^2^_1, 476_ = 0.941, p = 0.332). The population of the cross was not significant (GLM, χ^2^_1, 480_ = 0.015, p = 0.903). No other factors were significant (p > 0.248 in all cases).

None of the temperature factors significantly predicted refractory period for polyandry (GLM, p > 0.259 in all cases).The refractory period for pseudopolyandry was affected by a significant interaction between the population of the cross, the temperature the female was kept at and the temperature the mating occurred at (GLM, χ^2^_3, 45_ = 4.541, p = 0.033). All other factors were not significant (p > 0.436 in all cases). The total number of offspring produced tended to increase when the mating took place at a higher temperature, although this was non-significant (GLM, χ^2^_1, 476_ = 3.704, p = 0.054). If that factor is removed males kept at high temperatures sired more offspring (GLM, χ^2^_1, 477_ = 21.052, p < 0.001) while females kept at higher temperatures produced fewer offspring (GLM, χ^2^_1, 477_ = 12.783, p < 0.001). None of the interactions were significant for total offspring production (p > 0.250 in all cases).

### Hunger and remating

Few Greek females mated initially when starved, so they were removed from the analyses for remating behaviour (N = 17). Hunger state of the male and female were not significant factors for predicting rate of true polyandry (GLM: female: χ^2^_1, 351_ = 0.058, p = 0.810; male: χ^2^_1, 350_ < 0.001, p = 0.998), or the rate of pseudopolyandry (GLM, female: χ^2^_1, 348_ = 0.221, p = 0.639; male: χ^2^_1, 351_ = 0.653, p = 0.419. Population also did not affect polyandry (true polyandry: GLM, χ^2^_1, 348_ = 0.156, p = 0.693; pseudopolyandry: GLM, χ^2^_1, 350_ = 0.090, p = 0.765). All other factors were non-significant (polyandry: p > 0.200; pseudopolyandry: p > 0.553in all cases). Analysis of refractory period for polyandry was not possible due to lack of variation in refractory period for polyandrous females. No factors affected the refractory period for pseudopolyandry (p > 0.096 in all cases). Females on a low nutrient food produced fewer offspring (populations pooled, means of 73 and 33 offspring for fed and starved females respectively; GLM, χ^2^_1, 235_ = 81.141, p < 0.001). All other factors were non-significant for total offspring produced (p > 0.096 in all cases).

### Polyandry over time

**Figure 1:**
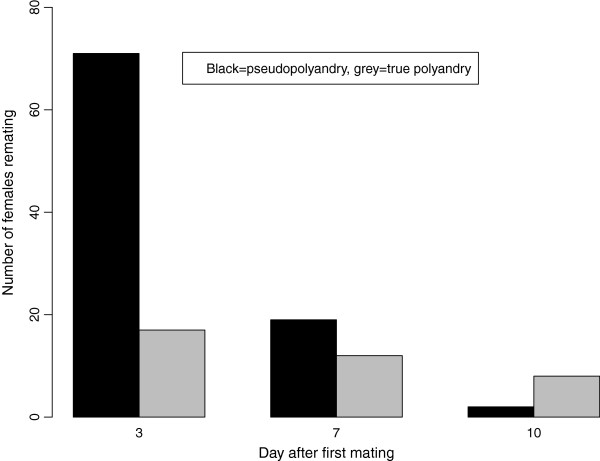
**Number of females remating at each opportunity.** Data from all remating experiments pooled, in total 794 mated females. Females that remated but never produced offspring are not included as their mating status could not be determined (N = 99).

Decreasing numbers of flies were pseudopolyandrous at each opportunity (Figure 1; all data pooled, 71/893, 19/805 and 2/795 remating at three, seven and ten days after the original mating respectively; Chi-squared test, χ^2^_2_ = 85.091, p < 0.001). All pairs were statistically significant (pair-wise Chi-squared tests, p < 0.001 in all cases). The rate of true polyandry did not change over time, (all data pooled, 17/893, 12/805 and 8/795 females showing true polyandry at three, seven and ten days after the mating respectively; Chi-squared test, χ^2^_2_ = 3.707, p = 0.157). The refractory period for pseudopolyandry was significantly shorter than the refractory period before polyandry (Figure 1, all data pooled; N = 129, medians of 3 and 7 days respectively, Mann–Whitney U-test, W = 1106.5, p < 0.001).

### Latency to mating and offspring production over all experiments

Latency before the original mating did not differ between females that went on to remate and those that never remated (all data pooled, log^10^ transformed, N = 893, Welch’s t-test, t_1, 208_ = −0.088, p = 0.930). Copulation duration of the original mating did not differ between females that went on to remate and those that never remated (all data pooled, square root transformed, N = 893, Welch’s t-test, t _1, 188_ = 0.617, p = 0.538). A significant difference was found in the total number of offspring produced between females of different mating status (means of 86, 64 and 70 offspring for monandrous, pseudopolyandrous and true polyandrous females respectively, N = 893, Kruskal-Wallis test, χ^2^_2_ =12.947, p = 0.002). Monandrous females produced significantly more offspring than those that were pseudopolyandrous (N = 757, Mann–Whitney U-test, W = 37279.5, p < 0.001). Polyandrous females produced a similar number of offspring to pseudopolyandrous females (N = 129, Mann–Whitney U-test, W = 1542.5, p = 0.408) and monandrous females (N = 702, Mann–Whitney U-test, W = 14013.0, p = 0.154). There was no difference in offspring production between the populations (all data pooled, medians of 79 & 69 for UK and Greek females respectively; Mann–Whitney U-test, W = 72429, p = 0.080).

### Male response to rivals

**Figure 2:**
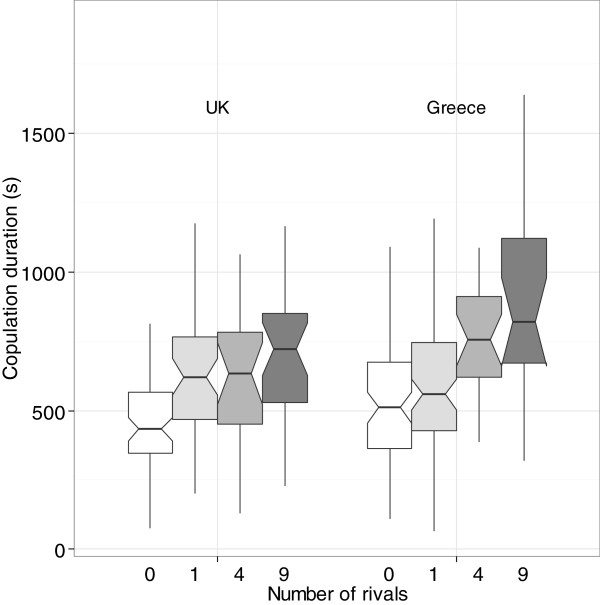
**Boxplot showing the copulation duration of UK and Greek flies with different numbers of rivals.** Flies were either kept alone (white), or exposed to one rival (light-grey), four rivals (medium-grey) or nine rivals (dark-grey) prior to mating. Plots indicate the median, interquartile range, and range. Notches indicate 95% confidence intervals [44].

In both UK and Greek populations, copulation duration varied significantly between different levels of male competition (Figure 2; GLM: UK, F_3, 163_ = 12.113, p <0.001; Greece, F_3, 192_ = 11.093, p < 0.001). However, the threshold of response varied between the two populations. The copulation duration of UK males exposed to any number of rivals (from one to nine rivals) was significantly higher than that of males kept alone prior to mating (Tukey post-hoc comparisons with lone male: vs. one rival, p < 0.005; vs. four rivals, p = 0.030; vs. nine rivals, p < 0.005). The copulation duration of Greek males was only increased when males were exposed to four or more rivals prior to mating (Tukey post-hoc comparisons with lone male: vs. one rival, p = 0.741; vs. four rivals, p < 0.005; vs. nine rivals, p < 0.005). These differences remain significant if the Bonferroni correction for multiple tests is used. The number of rivals a male was exposed to in pre-copulation treatments did not affect the subsequent offspring production of the female. This was true of both UK (Kruskal-Wallis: H = 3.27, p = 0.663, N = 69) and Greek (GLM: F_3, 53_ = 2.424, p = 0.076) flies.

## Discussion

As predicted by other studies [33-35], very few *D. subobscura* from either population remated. We found no evidence of a high willingness to remate in the Greek population, despite the report of frequent polyandry in the laboratory and field [36]. The majority of all rematings occurred when the female failed to produce offspring from her first mating. True polyandry was considerably rarer, with an overall rate of 4% over all experiments and no overall difference between the populations, although UK females showed an increase in likelihood of true polyandry when they were kept at higher temperatures. However, we did find a difference in the response of males to exposure to potential male rivals. The typical *Drosophila* response to rivals of increased copulation duration [35,37] was only shown by Greek males when exposed to four or more rivals, whereas the UK males responded to a single rival. This difference in response is the first evidence of intraspecific variation in this behaviour. However, as *D. subobscura* appears to be largely monandrous, this difference in response to rivals is unlikely to be related to the risk of sperm competition.

One potential inaccuracy in our methods is that we may have missed some females that showed true polyandry. If a female had a fertile first mating, but did not produce offspring before remating, she would be scored as showing pseudopolyandry. This is unlikely to be a major problem, because the females we used were seven days old, and should have been fully reproductively mature. Estimates of the survival of *D. subobscura*[45] and relatives [46,47] in the wild suggest that most flies die within ten days and few live more than three weeks. As a result, failing to oviposit for three days is likely to be costly to a female, and unlikely to be adaptive. We observed that overall 12% of monandrous females failed to produce offspring in the first three days but then went on to produce offspring. Hence at most 1.2% of females would have been wrongly assigned as pseudopolyandrous when they really showed true polyandry, increasing overall levels of true polyandry from 4 to 5.2%.

Although our results broadly concur with previous work that suggests *D. subobscura* is monandrous, discrepancies remain. Previous work by several research groups found no evidence of remating in *D. subobscura* at all [33-35] although Maynard Smith [33] did find that mated but uninseminated females were willing to remate (pseudopolyandry). The most likely explanation for this is that these studies all used small sample sizes (N <51 in all cases), so may have missed relatively rare remating events. In addition temperature may have played a role. Maynard Smith conducted his experiments at 18-20°C, and found no true polyandry [33], and our UK flies showed no true polyandry at 18°C. This demonstrates the importance of repeating studies in different conditions, as otherwise we would not have detected polyandry in the UK population. The high level of true polyandry reported by Loukas *et al.*[36] in both wild caught and laboratory flies is more difficult to explain. In our results, starvation does not affect the rate of true polyandry, but temperature does. Unfortunately Loukas *et al*. do not report the temperature at which their flies were maintained. The flies used in the experiment were the F_2_ progeny of wild flies trapped in August 1980 [36], so the experiment must have been carried out sometime between October and February. Hence a wide range of temperatures would be possible. Given that we observed higher rates of polyandry at both 18°C and 25°C, temperature may explain their laboratory results.

The high rate of true polyandry they detected in the wild caught flies is more difficult to explain. One possibility is that many of the wild caught females were old, and so had had more opportunities to remate. However, our experiment covered two and a half weeks, which should include the majority of most females’ lives [45]. Alternatively, females in nature may remate because they are exposed to a wider selection of males, and remate only when courted by very high quality males (“trading up”) [48]. However, our presentation of three males to each mated female makes it likely that at least one male would be superior to her original mate. In a similar vein, females in nature are likely to be harassed by males almost continuously when ovipositing (Price, pers. obs.). Females might remate in nature to reduce harassment, although this seems unlikely as females are often harassed by mutliple males, so mating with one will not help, and recently mated females are still harassed. It is also possible that the flies Loukas *et al*. used were different to ours. We collected from the same location which has been preserved as a nature reserve and has had a consistent ecology. Nevertheless, the 30 year gap between collections could have allowed an evolutionary change in mating system. Unfortunately, testing this would require access to the flies Loukas *et al.* used, and an in depth knowledge of the genetic basis of mating system in this species, neither of which is available at present. Finally, it is possible that a complex suite of factors such as temperature, length of reproductive season and condition of both males and females act together to increase rate of true polyandry in the Greek population, that cannot be easily replicated in the laboratory [22,25,49].

If we accept that our estimates of true polyandry are solid, then there is still the question of whether *D. subobscura* can be considered monandrous. Firstly, can a species showing up to 8% polyandry in controlled laboratory experiments be called monandrous? A strict definition of monandry, where no individuals ever remate, would be extremely difficult to prove for any species, and so would restrict the use of the category to theory. Furthermore, all animals make mistakes, and so even a species that has been strongly selected for monandry would be likely to remate in at least some unusual circumstances. In practice, there is an enormous amount of variation in which species are described as monandrous. Some authors describe a species where up to 25% of the females act polyandrously as a monandrous species [50]. Over all experiments, *D. subobscura* showed a four percent rate of true polyandry, but this varied between conditions, most strongly with temperature. This illustrates the difficulties of determining the mating system of a species from laboratory experiments. Overall, the four percent remating rate of *D. subobscura* probably does make it reasonable to call it monandrous [51]. However our laboratory estimates are still contradicted by Loukas *et al.*, who estimated 23% multiple paternity in nature. New measurements of polyandry in natural populations, using modern molecular techniques would be useful to resolve this.

The majority of rematings (71%) that we observed were pseudopolyandry. The rate of pseudopolyandry followed a very different pattern to that of true polyandry, showing a strong decrease in frequency with time. In serially true polyandrous species the propensity to remate is expected to increase over time [52,53], although in our experiments true polyandry remained constant over time. If females can detect that their first mating will not produce offspring, perhaps due to a failure of sperm transfer, male infertility, or genetic incompatibility, then remating as early as possible will increase her fitness. As predicted, in this study females that were pseudopolyandrous were far more likely to remate at their first opportunity than the second or third.

The contrasting impacts of both time and temperature on pseudopolyandry compared to true polyandry suggests that the two are indeed distinct behaviours, likely to be driven by different evolutionary pressures and controlled by different mechanisms. Moreover, the consequences of pseudopolyandry are likely to be far less wide ranging than those of true polyandry. The area where pseudopolyandry is likely to be most important is in disease transmission and epidemiology, where fertile matings are not required [54]. Pseudopolyandry is likely to be very widespread, with work from a wide variety of species showing that females are likely to remate if their first mating is infertile [49,55-60]. However, determining whether a species shows true or pseudopolyandry is likely to be difficult for many species, requiring extensive behavioural information on the species, best supported with molecular investigation of multiple paternity [61]. Genetic incompatibility provides a further complication, with some crosses between individuals likely to show greatly reduced offspring fitness, but not necessarily complete failure [17]. In addition, rates and drivers of both true and pseudopolyandry are likely to differ between populations, and between environments, as shown by some of the experiments presented here. Nevertheless, we believe that despite the difficulties of accurately determining the mating system, and potential complications due to interactions between individuals, the terms “true polyandry” and “pseudopolyandry” will add useful clarity to the field.

If *D. subobscura* is indeed a largely monandrous species, then why do males show an increase in copulation duration when exposed to potential mating rivals? The threat of sperm competition is likely to be very low for these males, even if the 23% true polyandry rate is true. One possibility is that males are responding to the likelihood of mating with more than one female. If females are monandrous, the chance of encountering a female that is willing to mate is lower when rivals are present. Hence males may maximise their fitness by investing all their reserves in the first female they mate with [62]. Alternatively, the response may not be adaptive, but might be due to conflict between males [63]. Longer copulation durations in *D. subobscura* may be due to exhaustion or damage in males exposed to rivals. However, we currently do not know why males from the two populations should show different patterns of response to rivals. All previous studies have found that all males respond to the presence of one or more rivals with the same increase in copulation duration [35,37,64,65]. The only exception is *D. bifasciata*, in which males show no response to rivals at all, irrespective of how many are present [66]. The difference between the populations in their response to rivals, with no corresponding difference in female willingness to remate, suggests that this response is not related to sperm competition in this species. Copulation duration and amount of sperm transferred are not necessarily related in *D. melanogaster* either [39,67]. What does drive the difference in male response in *D. subobscura* is currently unknown. There are a great many possible ecological differences between the UK and Greek populations. For example, population density may differ between populations, causing different rates of male-male contact and aggression [68]. Our present paucity of information about the two populations makes it difficult to identify the cause, now that it is unlikely to be sperm competition.

## Conclusions

Overall, our results suggest that *D. subobscura* is a generally monandrous species, although further estimates of polyandry in natural populations would be useful to confirm this. Previous differences in estimates of polyandry were probably driven by differences in the methods used to assay remating, compounded by small sample sizes. However, our two populations did differ in the response males shown to exposure to potential rivals, making it unlikely that this response is related to the risk of sperm competition, as predicted by previous studies. As found in other species, some female *D. subobscura* remate after a mating that failed to fertilise them (pseudopolyandry), while only a small proportion exhibit true polyandry. We suggest that this distinction between true polyandry and pseudopolyandry is one that is worth making in future studies of female remating behaviour, especially when conditions may affect the two behaviours differently.

## Methods

### Experimental populations

We collected females of *D. subobscura* from Adel Dam, Leeds, UK (53°52′1.30″N, 1°35′7.83″E) in May 2011 (“UK” flies) and from Parnitha National Park, Athens, Greece (38°10′24.24″N, 23°43′2.64″E) in April 2011 (“Greek” flies). Flies were maintained as mixed populations in the laboratory (N = 200 per generation), descended from 24 and 27 wild females for the UK and Greek populations respectively. We maintained them on a maize, sugar, agar and yeast medium at 21°C [42] with a 14:10 hr photoperiod (lights on at 10:00 GMT). We conducted all experiments in these conditions unless stated otherwise.

### Population differences in remating

To examine the willingness of females from both populations to remate, we first mated virgin females to males, and then presented them with regular opportunities to mate with another male. For each population we collected flies as one day old virgins under CO_2_ anaesthesia and transferred them to single sex groups of 15–20 flies per vial. We mated all flies at eight days old, by which time *D. subobscura* are sexually mature [34] and unaffected by the prior CO_2_ anaesthesia [69]. At seven days old we aspirated each female into an individual vial. This increases mating propensity in females as they acclimatise to the vial [69]. Males were also placed in a vial on their own one day before they would be used in a mating, which improves their chance of mating [33], although the mechanism for this is unclear. We began each mating and remating experiment within 15 minutes of the light period of our photoperiod beginning. This provides a “dawn” stimulus, when *D. subobscura* are most active in nature [70]. We aspirated a male into a vial containing a single female, and observed the pair for two hours, recording the time to start of mating (latency) and copulation duration. A period of two hours was used for several reasons. Firstly, 88% of the *D. subobscura* that mate within two hours mate within the first 60 minutes. Secondly*, D. subobscura* are thought to be most active for a few hours just after dawn and before dusk, making two hours a reasonable estimate of an interaction period. Once a mating ended we discarded the male. For the first matings, we discarded any females that were unmated after two hours. A mean of 56% females failed to mate at the first opportunity, and this ranged from 32% to 86% for different experiments. This low mating rate by virgin *D. subobscura* is not unusual for the species, having been reported in previous experiments [43]. However, it does mean that the females that did mate are unlikely to be an entirely random sample of females, particularly in the female starved treatments where very few females mated. It is possible that females that did not remate on day 8 would have mated later if given the opportunity, and might show different remating behaviour to the initially mated females. Our experimental design cannot directly address this, although we are not aware of any previous work that has suggested such changes are likely in *Drosophila*. Once mated, we then gave females remating opportunities with new seven day old virgin males on three occasions, at three, seven and ten days after the original mating. The remating opportunity lasted two hours, after which we discarded the males.

We moved all females into new vials with fresh food three days after the original mating opportunity, immediately after the second mating opportunity, and ten days after the original mating, immediately after the final remating opportunity. *Drosophila* of the Obscura group show an insemination reaction on mating in which the ejaculate becomes a gelatinous mass for approximately 30 minutes after mating, and sperm cannot be used by the female to fertilise eggs [71]. Hence any offspring in the first vial cannot have been fathered by the second male, and would indicate that the first mating was fertile. We discarded the females after 17 days, and we monitored the vials they were kept in for emerging adults. One week after the majority of vials in a condition had the first adult emerge, we counted the total number of adult offspring in each vial. Females that remated after producing offspring were recorded as showing true polyandry. If a remating occurred before the female produced any offspring we termed it pseudopolyandry. All females were mated only to males from the same population.

### Examination of which sex controls remating

To determine whether the possible differences between populations in remating rate were male or female controlled, we repeated the experiment above, but initially mated half the females with a male from the same population, and half with a male from the other population Remating opportunities were with males of alternating populations. This was the only experiment that crossed males and females of different populations. All subsequent experiments crossed males and females from the same population.

### Effect of temperature on remating

To detect any effect of temperature on remating behaviour, we repeated the original design independently at 18°C and 25°C. We collected virgins from vials maintained at 21°C as above, but kept the adults at either 18°C or 25°C. We carried out the matings at the temperature the males and females were raised at i.e. 18°C or 25°C. Crosses with adult flies kept at 18°C or 25°C were also performed at 21°C, to control for the temperature during courtship/mating, which can affect the degree of remating [72]. To establish whether any effect of temperature was male or female controlled, we kept females at 18°C and mated them with males from 25°C, and we mated females kept at 25°C with males from 18°C. The matings for these crosses occurred at 21°C. All remating opportunities were with males raised at the same temperature as the male in the original mating and occurred at the same temperature as the original mating.

### Effect of manipulating hunger state

To determine whether flies in a worse condition had altered mating and remating behaviour, we altered the diet of males and females. In order to starve the females we kept them on food containing 1/5 of the yeast of the standard food from collection as one day old virgins for their entire adult lives. This degree of starvation altered remating behaviour in *D. melanogaster*[25]. To starve the males we aspirated them at six days old into an individual vial containing a piece of blue paper over damp cotton wool, providing water but no nutrition [43]. We mated the flies at seven days old. We crossed females, either starved or in normal condition, with males, in starved or normal condition. All remating opportunities were with males in normal condition (not starved).

### Male response to rivals

To examine the response of males to exposure to potential mating rivals, we placed one day old virgin males either alone or with one, four or nine rival males. Males were kept in these conditions until they were transferred to the female vial for mating. A single male was used from each vial to avoid pseudoreplication [35], and males from vials containing dead males were not used. Virgin females were collected as above. As standard, we recorded the occurrence of mating, latency and duration of copulation. Each pair was allowed two hours to mate, after which we discarded males. As in the first experiment, mated females were then retained for offspring counts. We repeated this process independently for both Greek and UK populations.

### Data analysis

Data were analysed using IBM SPSS v20 and R 2.15.1 [73]. Females that remated but produced no offspring at all were excluded from analyses, as the female might have been infertile; in which case determining whether she showed true polyandry or pseudopolyandry was not possible. Where Generalised Linear Models were used, we constructed the maximal model and then removed non-significant factors in a stepwise manner.

## Competing interests

The authors declare they have no competing interests.

## Authors’ contributions

DF & TP conceived the remating experiments, RD & TP conceived the response to rivals experiments. DF participated in all experiments, performed statistical analysis for the remating experiments and drafted the manuscript. RD participated in all experiments, performed statistical analysis for the response to rivals experiments and drafted the manuscript. TP participated in some of the experiments, guided statistical analysis and drafted the manuscript. All authors read and approved the final manuscript.

## References

[B1] Arnqvist G Nilsson T The evolution of polyandry: multiple mating and female fitness in insects Anim Behav 2000 60 2 145 164 10.1006/anbe.2000.1446 10973716

[B2] Simmons LW The evolution of polyandry: an examination of the genetic incompatability and good-sperm hypotheses J Evol Biol 2001 14 585 594 10.1046/j.1420-9101.2001.00309.x

[B3] Holman L Kokko H The consequences of polyandry for population viability, extinction risk and conservation Phil Trans R Soc B 2013 368 20120053 10.1098/rstb.2012.0053 23339244 PMC3576587

[B4] Jaffé R Moritz RFA Kraus FB Gene flow is maintained by polyandry and male dispersal in the army ant *Eciton burchellii* Popul Ecol 2009 51 227 236 10.1007/s10144-008-0133-1

[B5] Sherman PW Seeley TD Reeve HK Parasites, pathogens and polyandry in social hymenoptera Am Nat 1988 131 4 602 610 10.1086/284809 18811329

[B6] Poiani A Wilks C Sexually transmitted diseases: a possible cost of promiscuity in birds Auk 2000 117 4 1061 1065

[B7] Price TA Hurst GD Wedell N Polyandry prevents extinction Current biology : CB 2010 20 5 471 475 10.1016/j.cub.2010.01.050 20188561

[B8] Trivers RL Parent-offspring conflict Amer Zool 1974 14 249 264

[B9] Hughes WO Oldroyd BP Beekman M Ratnieks FL Ancestral monogamy shows kin selection is key to the evolution of eusociality Science 2008 320 5880 1213 1216 10.1126/science.1156108 18511689

[B10] Thornhill R Cryptic female choice and its implications in the scorpionfly *Harpobittacs nigriceps* Am Nat 1983 122 6 765 788 10.1086/284170

[B11] Parker GA Sperm competition and its evolutionary consequences in the insects Biol Rev 1970 45 525 567 10.1111/j.1469-185X.1970.tb01176.x

[B12] Hosken DJ Stockley P Tregenza T Wedell N Monogamy and the battle of the sexes Annu Rev Entomol 2009 54 361 378 10.1146/annurev.ento.54.110807.090608 18793102

[B13] Rowe L Arnqvist G Sih A Krupa JJ Sexual conflict and the evolutionary ecology of mating patterns: water striders as a model system Trends Ecol Evol 1994 9 289 293 10.1016/0169-5347(94)90032-9 21236857

[B14] Parker GA Sexual conflict over mating and fertilization: an overview Philos Trans R Soc Lond B Biol Sci 2006 361 1466 235 259 10.1098/rstb.2005.1785 16612884 PMC1569603

[B15] Mossinson S Yuval B Regulation of sexual receptivity of female Mediterranean fruit flies: old hypotheses revisited and a new synthesis proposed J Insect Physiol 2003 49 6 561 567 10.1016/S0022-1910(03)00027-1 12804715

[B16] Zeh JA Zeh DW The evolution of polyandry I: intragenomic conflict and genetic incompatability Proceedings of the Royal Society B: Biological Sciences 1996 263 1377 1711 1717 10.1098/rspb.1996.0250

[B17] Zeh JA Zeh DW The evolution of polyandry II: post-copulatory defences against genetic incompatability Proceedings of the Royal Society B: Biological Sciences 1997 264 69 75 10.1098/rspb.1997.0010

[B18] Kvarnemo C Simmons LW Polyandry as a mediator of sexual selection before and after mating Philos Trans R Soc Lond B Biol Sci 2013 368 1613 20120042 10.1098/rstb.2012.0042 23339234 PMC3576577

[B19] Westneat DF Extra-pair copulations in a predominately monogamous birds: observations of behaviour Anim Behav 1987 35 865 876 10.1016/S0003-3472(87)80122-7

[B20] Haddrill PR Shuker DM Amos W Majerus ME Mayes S Female multiple mating in wild and laboratory populations of the two-spot ladybird, Adalia bipunctata Mol Ecol 2008 17 13 3189 3197 10.1111/j.1365-294X.2008.03812.x 18522693

[B21] Simmons LW Sperm competition and its evolutionary consequences in the insects 2001 Princeton, New Jersey: Princeton University Press

[B22] Välimäki P Kaitala A Kokko H Temporal patterns in reproduction may explain variationin mating frequencies in the green-veined white butterfly Pieris napi Behav Ecol Sociobiol 2006 61 1 99 107 10.1007/s00265-006-0240-y

[B23] McFarlane SE Lane JE Taylor RW Gorrell JC Coltman DW Humphries MM Boutin S McAdam AG The heritability of multiple male mating in a promiscuous mammal Biol Lett 2011 7 3 368 371 10.1098/rsbl.2010.1003 21159688 PMC3097859

[B24] Syriatowicz A Brooks R Sexual responsiveness is condition-dependent in female guppies, but preference functions are not BMC Ecol 2004 4 5 10.1186/1472-6785-4-5 15117410 PMC411045

[B25] Fricke C Bretman A Chapman T Female nutritional status determines the magnitude and sign of responses to a male ejaculate signal in Drosophila melanogaster J Evol Biol 2010 23 1 157 165 10.1111/j.1420-9101.2009.01882.x 19888937

[B26] Välimäki P Kaitala A Does a lack of mating opportunites explain monandry in the green-veined white butterly (*Pieris napi*)? Oikos 2006 115 110 116 10.1111/j.2006.0030-1299.14947.x

[B27] Harano T Miyatake T Heritable variation in polyandry in *Callosobruchus chinensis* Anim Behav 2005 70 2 299 304 10.1016/j.anbehav.2004.10.023

[B28] Price TAR Lewis Z Smith DT Hurst GDD Wedell N Remating in the laboratory reflects rates of polyandry in the wild Anim Behav 2011 82 6 1381 1386 10.1016/j.anbehav.2011.09.022

[B29] Latorre A Moya A Ayala FJ Evolution of mitrochondrial DNA in *Drosophila subobscura* Proc Natl Acad Sci USA 1986 83 8649 8653 10.1073/pnas.83.22.8649 16578796 PMC386988

[B30] Huey RB Gilchrist GW Carlson ML Berrigan D Serra L Rapid evolution of a geographic cline in size in an introduced fly Science 2000 287 308 309 10.1126/science.287.5451.308 10634786

[B31] Davis JR Jenkinson LS Lawton JH Shorrocks B Wood S Making mistakes when predicting shifts in species range in response to global warming Nature 1998 391 783 786 10.1038/35842 9486646

[B32] Balanya J Oller JM Huey RB Gilchrist GW Serra L Global genetic change tracks global climate warming in Drosophila subobscura Science 2006 313 5794 1773 1775 10.1126/science.1131002 16946033

[B33] Maynard Smith J Fertility, mating behaviour and sexual selection in *Drosophila subobscura* J Genet 1956 54 2 261 279 10.1007/BF02982781 15876580

[B34] Holman L Freckleton RP Snook RR What use is an infertile sperm? A comparative study of sperm-heteromorphic drosophila Evolution 2008 62 2 374 385 10.1111/j.1558-5646.2007.00280.x 18053077

[B35] Lizé A Doff RJ Smaller EA Lewis Z Hurst GD Perception of male-male competition influences *Drosophila* copulation behaviour even in species where females rarely remate Biol Lett 2012 8 1 35 38 10.1098/rsbl.2011.0544 21752815 PMC3259955

[B36] Loukas M Vergini Y Krimbas CB The genetics of *Drosophila subobscura* populatios XVIII. Multiple insemination and sperm displacement in Drosophila subobscura Genetica 1981 57 29 37 10.1007/BF00057540

[B37] Bretman A Fricke C Chapman T Plastic responses of male Drosophila melanogaster to the level of sperm competition increase male reproductive fitness Proceedings Biological sciences / The Royal Society 2009 276 1662 1705 1711 10.1098/rspb.2008.1878 19324834 PMC2660996

[B38] Price TAR Lizé A Marcello M Bretman A Experience of mating rivals causes males to modulate sperm transfer in the fly *Drosophila pseudoobscura* J Insect Physiol 2012 58 1669 1675 10.1016/j.jinsphys.2012.10.008 23085556

[B39] Garbaczewska M Billeter JC Levine JD Drosophila melanogaster males increase the number of sperm in their ejaculate when perceiving rival males J Insect Physiol 2013 59 3 306 310 10.1016/j.jinsphys.2012.08.016 23178803

[B40] Wigby S Sirot LK Linklater JR Buehner N Calboli FC Bretman A Wolfner MF Chapman T Seminal fluid protein allocation and male reproductive success Current biology: CB 2009 19 9 751 757 10.1016/j.cub.2009.03.036 19361995 PMC2737339

[B41] Fedorka KM Winterhalter WE Ware B Perceived sperm competition intensity influences seminal fluid protein production prior to courtship and mating Evolution 2011 65 2 584 590 10.1111/j.1558-5646.2010.01141.x 21271997

[B42] Steele RH Courtship feeding in *Drosophila subobscura.* I. The nutritional significance of courtship feeding Anim Behav 1986 34 1087 1098 10.1016/S0003-3472(86)80168-3

[B43] Immonen E Hoikkala A Kazem AJN Ritchie MG When are vomiting males attractive? Sexual selection on condition-dependent nuptial feeding in Drosophila subobscura Behav Ecol 2009 20 2 289 295 10.1093/beheco/arp008

[B44] McGill R Tukey JW Larsen WA Variations of box plots Am Stat 1978 32 1 12 16

[B45] Rosewell J Shorrocks B The implication of survival rates in natural populations of *Drosophila*: capture-recapture experiments on domestic speceis Biol J Linn Soc 1987 32 373 384 10.1111/j.1095-8312.1987.tb00438.x

[B46] Powell J Progress and prospects in evolutionary biology: the Drosophila model 1997 Oxford: Oxford University Press

[B47] Dobzhansky T Wright S Genetics of natural populations X. Dispersion rates in *Drosophila pseudoobscura* Genetics 1943 28 304 340 17247091 10.1093/genetics/28.4.304 PMC1209213

[B48] Halliday TR Bateson P The study of mate choice Mate choice 1983 Cambridge: Cambridge University Press

[B49] Gavriel S Gazit Y Yuval B Remating by female Mediterranean fruit flies (Ceratitis capitata, Diptera: Tephritidae): temporal patterns and modulation by male condition J Insect Physiol 2009 55 7 637 642 10.1016/j.jinsphys.2009.04.002 19482138

[B50] Torres-Vila LM Rodriguez-Molina MC Jennions MD Polyandry and fecundity in the Lepidoptera: can methodological and conceptual approaches bias outcomes? Behav Ecol Sociobiol 2004 55 4 315 324 10.1007/s00265-003-0712-2

[B51] Arnqvist G Comparative evidence for the evolution of genitalia by sexual selection Nature 1998 393 784 786 10.1038/31689

[B52] Thornhill R Alcock J The evolution of insect mating systems 1983 Cambridge: Harvard University Press

[B53] Eberhard WG Female control: sexual selection by cryptic female choice 1996 Princeton: Princeton University Press

[B54] Ashby B Gupta S Sexually transmitted infections in polygamous mating systems Phil Trans R Soc B 2013 368 20120048 10.1098/rstb.2012.0048 23339239 PMC3576582

[B55] Xue L Noll M Drosophila female sexual behavior induced by sterile males showing copulation complementation Proc Natl Acad Sci USA 2000 97 7 3272 3275 10.1073/pnas.97.7.3272 10725377 PMC16228

[B56] Katiyar KP Ramirez E Mating frequency and fertility of mediterranean fruit fly females alternately mated with normal and irradiated males J Econ Entomol 1970 63 4 1248 1250

[B57] Chapman T Miyatake T Smith HK Partridge L Interactions of mating, egg production and death rates in females of the Mediterranean fruit fly, Ceratitis capitata Proceedings Biological sciences / The Royal Society 1998 265 1408 1879 1894 10.1098/rspb.1998.0516 9802244 PMC1689375

[B58] Shelley TE Kennelly S Influence of male diet on male mating success and longevity and female remating in the Medeterranean fruit fly (Diptera: Tephritidae) under laboratory conditions Florida Entomol 2002 85 4 572 579 10.1653/0015-4040(2002)085[0572:IOMDOM]2.0.CO;2

[B59] Proshold FI Remating by Gypsy Moths (Lepidoptera: Lymantriidae) Mated wth F1-Sterile Males as a Function of Sperm Within the Spermatheca J Econ Entomol 1995 88 3 644 648

[B60] Taylor OR Relationship of Multiple mating to Fertility in *Atteva punctella* (Lepidoptera: Yponomeutidae) Ann Entomol Soc Am 1967 60 3 583 590

[B61] Bretman A Tregenza T Measuring polyandry in wild populations: a case study using promiscuous crickets Mol Ecol 2005 14 7 2169 2179 10.1111/j.1365-294X.2005.02556.x 15910335

[B62] Kokko H Jennions MD Parental investment, sexual selection and sex ratios J Evol Biol 2008 21 4 919 948 10.1111/j.1420-9101.2008.01540.x 18462318

[B63] Chen S Lee AY Bowens NM Huber R Kravitz EA Fighting fruit flies: a model system for the study of aggression Proc Natl Acad Sci USA 2002 99 8 5664 5668 10.1073/pnas.082102599 11960020 PMC122828

[B64] Bretman A Fricke C Hetherington P Stone R Chapman T Exposure to rivals and plastic responses to sperm competition in Drosophila melanogaster Behav Ecol 2010 21 2 317 321 10.1093/beheco/arp189

[B65] Bretman A Gage MJ Chapman T Quick-change artists: male plastic behavioural responses to rivals Trends Ecol Evol 2011 26 9 467 473 10.1016/j.tree.2011.05.002 21680050

[B66] Lizé A Price TAR Marcello M Smaller EA Lewis Z Hurst GDD Males do not prolong copulation in response to competitor males in the polyandrous fly *Drosophila bifasciata* Physiol Entomol 2012 37 3 227 232 10.1111/j.1365-3032.2012.00836.x

[B67] Gilchrist AS Partridge L Why it is difficult to model sperm displacement in *Drosophila melanogaster*: the realtionship between sperm transfer and copulation duration Evolution 2000 54 2 534 542 10937230 10.1111/j.0014-3820.2000.tb00056.x

[B68] Cushing BS Razzoli M Murphy AZ Epperson PM Le WW Hoffman GE Intraspecific variation in estrogen receptor alpha and the expression of male sociosexual behavior in two populations of prairie voles Brain Res 2004 1016 2 247 254 10.1016/j.brainres.2004.05.010 15246861

[B69] Barron AB Anaesthitising *Drosophila* for behavioural studies J Insect Physiol 2000 46 439 442 10.1016/S0022-1910(99)00129-8 12770207

[B70] Shorrocks B Charlesworth P The distribution and abundance of the British fungal breeding *Drosophila* Ecol Entomol 1980 5 61 78 10.1111/j.1365-2311.1980.tb01124.x

[B71] Wheeler NR The insemination reaction in intraspecific matings of Drosophila Univ Texas Publ 1947 4720 78 115

[B72] Best AR Lewis Z Hurst GDD Lizé A Thermal environment during and outside courtship jointly determine female remating rate in *Drosophila melanogaster* Anim Behav 2012 83 6 1483 1490 10.1016/j.anbehav.2012.03.022

[B73] Ihaka R Gentleman R R: A language for data analysis and graphics J Comput Graph Stat 1996 5 3 299 314

